# Chromogranin A mediates glucocorticoid-induced enhancement of vesicular dopamine storage and secretion in PC12 cells

**DOI:** 10.1016/j.jbc.2026.113460

**Published:** 2026-08-17

**Authors:** Noemí Socas-Pérez, Ayoze González-Santana, Judith Estévez-Herrera, Natalia Domínguez, Marcial Camacho, Ricardo Borges, José David Machado

**Affiliations:** 1 Departamento de Medicina Física y Farmacología, Facultad de Medicina, Universidad de La Laguna, La Laguna, Tenerife, Spain; 2 Departamento de Bioquímica, Microbiología, Biología Celular y Genética, Facultad de Ciencias, Universidad de La Laguna, La Laguna, Tenerife, Spain

**Keywords:** chromogranin A, dopamine, exocytosis, glucocorticoids, neurotransmitters, secretion, quantal release

## Abstract

Biogenic amines, including catecholamines, are concentrated within large dense-core vesicles through interactions with intravesicular matrix proteins and ATP. Efficient accumulation also depends on additional accessory components that regulate vesicular storage and release. Glucocorticoids are known to enhance catecholamine secretion and quantal size in PC12 cells, although the molecular mechanisms linking these effects to vesicular dopamine storage remain incompletely understood. Glucocorticoid treatment upregulated key enzymes involved in catecholamine biosynthesis, most notably tyrosine hydroxylase, while also enhancing the expression of chromogranin A. These molecular adaptations were postulated to increase vesicular dopamine content and improve secretion efficiency. To determine how the observed molecular alterations translated into functional changes, we quantified transcriptional and protein level regulation of key targets using qRT-PCR and western blotting. We then linked these molecular effects to cellular function by measuring intracellular dopamine content *via* high-performance liquid chromatography, monitoring stimulus-evoked secretion with online secretion assays and characterizing quantal exocytosis at single-cell resolution using carbon-fiber amperometry. Corticosteroids enhance dopamine storage within secretory vesicles in a time dependent manner, likely by promoting matrix formation. Our results indicate that dopamine accumulation is limited by vesicular storage capacity rather than synthesis alone in PC12 cells. Furthermore, combining glucocorticoid treatment with L-DOPA to increase amine availability produces a marked increase in vesicular dopamine content and a robust augmentation of stimulus-evoked secretion. Collectively, these findings identify chromogranin A-dependent secretory vesicle matrix formation, temporally coupled to dopamine loading, as a previously underappreciated mechanism by which glucocorticoids regulate vesicular dopamine storage and secretory efficiency.

Neurosecretion is the finely orchestrated calcium dependent process by which neurons release neurotransmitters by synaptic vesicles and neuropeptides and amines by large dense-core vesicle (LDCVs) through regulated exocytosis. In this process, secretory vesicles fuse with the plasma membrane in response to membrane depolarization, ensuring rapid and precise signal transmission essential for proper nervous system function (1, 2). A key determinant of vesicle release efficacy is the concentration of transmitters stored within vesicles. High vesicular content underpins a fast, reliable and robust communication system (3, 4, 5, 6); even modest alterations in the amount of neurotransmitter per vesicle can significantly affect the duration and strength of receptor activation (7).

Catecholamines are sequestered in LDCVs at concentrations reaching 0.8 to 1 M, as revealed by patch-amperometry studies in chromaffin cells (8, 9). To maintain these high concentrations and reduce the osmotic strength, catecholamines interact with intravesicular chromogranins and ATP. The critical role of chromogranins is underscored by findings that their absence leads to diminished transmission efficiency (8, 10).

In chromaffin cells, dopaminergic neurons and in rat pheochromocytoma cell line PC12, administration of the catecholamine precursor L-DOPA elevates vesicular dopamine (DA) (8, 10, 11, 12, 13, 14, 15, 16, 17, 18). Conversely, approaches that inhibit key processes, such as tyrosine hydroxylase (TH) activity (16), the maintenance of the pH/ψ gradient *via* V-ATPase inhibition (19) or the function or expression of SLC18A2 (16) and nucleotide transporters (20, 21), lead to reduced vesicular content. However, not all cells accumulate catecholamines with the same performance. Therefore, native chromaffin cells typically exhibit quantal sizes on the order of 1 pC, corresponding approximately 3·10^6^ catecholamine molecules per vesicle (∼5000 zmol) (22, 23), while in PC12 cells the quantal size is ∼20 to 30 fC range, corresponding to approximately 6 to 9·10^5^ molecules of DA per vesicle (∼100–150 zmol) (12, 18). This lower dopamine storage capacity in PC12 cells likely reflects differences in the underlying mechanisms of amine synthesis and DA vesicular accumulation (24). While all biogenic amines are concentrated in LDCVs *via* their association with an intravesicular matrix, the proportion of matrix-bound *versus* free amines and their spatial distribution within the vesicle varies between cell types (25). Under resting conditions, approximately 90% of catecholamines are matrix-associated (26, 27), leaving a small free pool that appears as a “halo” by electron microscopy (28). Importantly, when cells are preloaded with L-DOPA, most newly synthesized DA preferentially localizes to this halo, prompting two key questions: (i) Can the amine cargo in PC12 cells be further enhanced beyond baseline levels and (ii) How sustained are the changes induced by L-DOPA preloading?

This study aims to explore pharmacological approaches that enhance biogenic amines storage efficiency within LDCVs, such as DA. Glucocorticoids have long been recognized as major regulators of the catecholaminergic phenotype in chromaffin and PC12 cells (29, 30), increasing catecholamine synthesis and secretion through the coordinated regulation of multiple genes involved in catecholamine biosynthesis, and secretion (24, 31, 32). Although these functional effects have been well documented, the molecular mechanisms by which glucocorticoids enhance vesicular catecholamine storage remain incompletely understood. By investigating the impact of glucocorticoid treatment on chromogranin A-dependent secretory vesicle matrix formation in parallel with strategies that optimize dopamine availability, we sought to identify the mechanisms coordinating vesicular dopamine loading and storage. Our findings highlight potential avenues for modulating vesicular storage capacity, which may inform the development of improved pharmacological strategies for dopamine related diseases.

## Results

### Temporal dynamic of dopamine uptake

**Figure 1:**
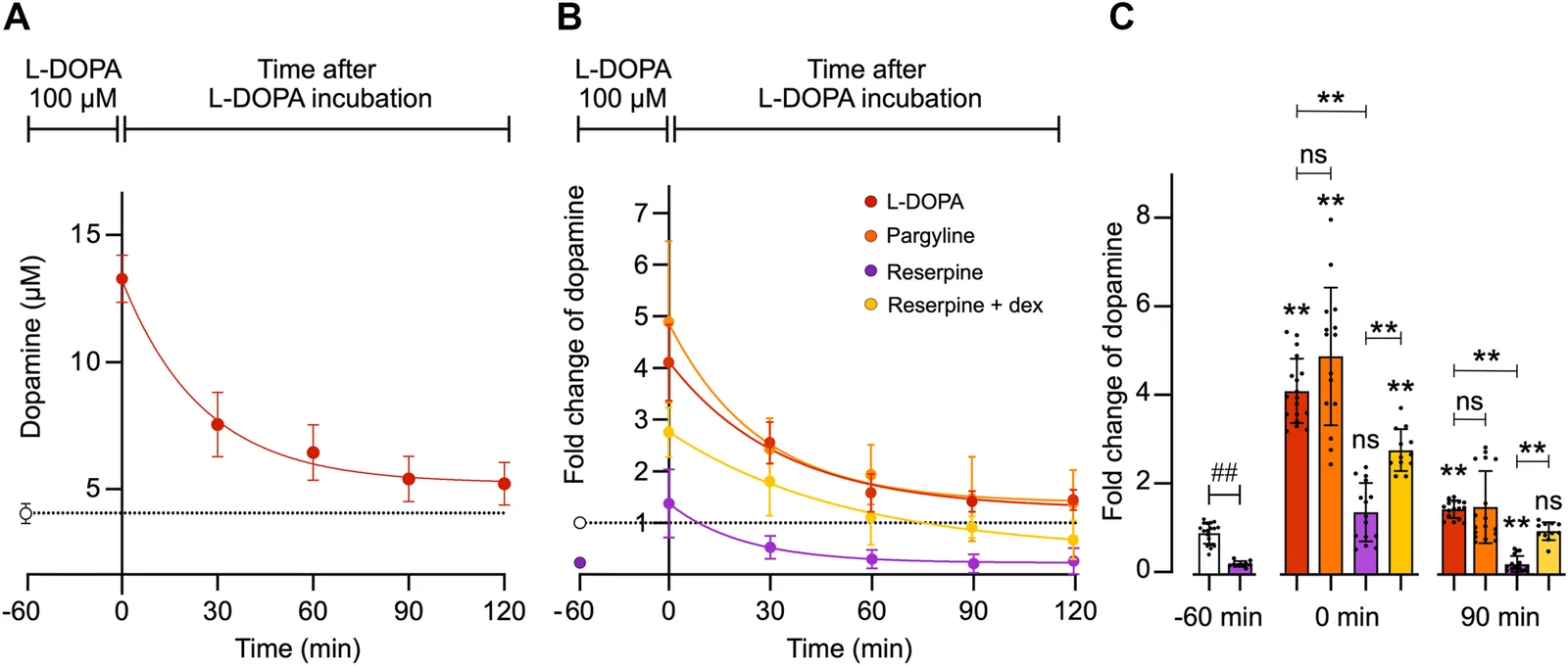
**Accessing dopamine uptake in PC12 cells.***A,* basal dopamine content and temporal dynamic of vesicular dopamine uptake. PC12 cells were incubated with 100 μM L-DOPA for 60 min, washed twice, and maintained in RPMI-1640 medium for 30-, 60-, 90-, and 120-min. Cells were then lysed, and total dopamine was quantified using HPLC-ED. *Data points* represent the mean ± SEM (n = 3; each experiment included six biological replicates). The dashed horizontal line denotes basal dopamine levels. The curve fit follows a single exponential decay with a tau of approximately 25 min. *B,* temporal dynamics of normalized DA. Each value was normalized by dividing it by the mean of the control group. DA levels were assessed after 60 min of L-DOPA (100 μM) *(red)* treatment, or 90 min of reserpine (10 μM) exposure *(violet)*, reserpine + DEX *(yellow)*, pargyline (10 μM) *(orange)* and at 30-, 60-, 90-, and 120-min post-L-DOPA incubation. Data points represent the mean ± SD (singles spots represent; n = 18 independent biological replicates per condition, collected across three independent experimental days) *(C)* basal DA content at −60 (white bar indicate DA content for control, L-DOPA and pargyline treatment), and after L-DOPA treatment (0 and 90 min) in PC12 cells with or without reserpine, reserpine + DEX or pargyline. *Bars* represent normalized data as means ± SD, singles spots represent; n = 18 independent biological replicates per condition, collected across three independent experimental days. Statistical significance was assessed by two-way ANOVA followed by Tukey's *post hoc* multiple-comparisons test (∗*p* < 0.05 and ∗∗*p* < 0.01), for comparing basal condition indicated by hashtag (##*p* < 0.01). DA, dopamine; DEX, dexamethasone.

A transient increase in vesicular dopamine content can be achieved by incubating cells with the synthetic precursor L-DOPA. To evaluate vesicular DA loading in PC12 cells, we first quantified the basal DA content by HPLC-ED, which reached approximately 3.8 μM (Fig. 1*A*). Importantly, this basal value, measured in 3 × 10^5^ cells, represents the total dopamine content present at the onset of the experiment and should not be interpreted as a cytosolic concentration. This measurement served as the reference point for normalizing and comparing all subsequent experimental conditions. Following baseline determination, PC12 cells were incubated with 100 μM L-DOPA for 60 min, reaching the total DA concentration of approximately 13 μM (Fig. 1*A*), which correspond to a 4.2-fold increase relative to basal level (Fig. 1*B*). The increase of DA followed an exponential increase with a time constant of approximately 30 min (data non shown), consistent with previous reports in our lab and another (11, 18). Subsequently, DA levels gradually decline following a one-phase exponential decay, characterized by a time constant (τ ∼ 25 min), stabilizing at about 1.3-fold over basal values around 90 min time points (Fig. 1, *A* and *B*). This data point likely reflects the time dependent uptake and store of DA into secretory vesicles. To confirm that DA was predominantly stored in secretory vesicles, SLC18A2 was inhibited using reserpine (10 μM) for 90 min prior to L-DOPA incubation. This treatment resulted in a near complete depletion of basal vesicular DA levels (Fig. 1*B*). In these reserpine-treated cells, subsequent incubation with L-DOPA for 60 min markedly attenuated DA accumulation, yielding only a 1.6-fold increase in DA levels (Fig. 1*B*). Thereafter, DA levels declined progressively following an exponential decay with τ ≈ 25 min, stabilizing at values close to the detection limit (Fig. 1*B*). A similar pattern was observed in DEX pretreated cells exposed to reserpine, where the absolute increase in DA driven by subsequent L-DOPA incubation was +1.7-fold (Fig. 1*C*). Pargyline (an irreversible inhibitor of both MAO-A and MAO-B isoforms) is known to increase cytosolic DA (33, 34). To test the contribution of MAOs we incubated cells for 60 min with 10 μM pargyline, and then cells were treated with L-DOPA and pargyline during 60 min. This combination raised the DA concentration to ∼5.2-fold, unveiling the contribution of cytosolic DA metabolism (Fig. 1, *B* and *C*). When both drugs were removed, DA content dropped following an exponential decay with a time constant of ∼24 min. After 30 min, DA content closely resembled those observed with L-DOPA alone. Based on this temporal profile, statistical analyses were performed at three key time points: −60 min, representing DA levels prior to the start of the experiment; 0 min, corresponding to peak dopamine accumulation across both cytosolic and LDCVs; and 90 min, when DA is preferentially stored within LDCVs. Overall, these results indicate that a fraction of the dopamine synthesized immediately after L-DOPA exposure remains transiently in the cytosol before being gradually sequestered into secretory vesicles. By 60 to 90 min, however, the majority of newly synthesized DA is efficiently compartmentalized into vesicles. Notably, in dexamethasone (DEX) pretreated cells exposed to reserpine, vesicular DA storage remained enhanced relative to reserpine alone (Fig. 1*C*), indicating a greater capacity to retain DA within secretory vesicles despite pharmacological blockade of SLC18A2-mediated uptake. Together, these results reflect a time-dependent shift toward predominant vesicular uptake, which is further amplified by glucocorticoid pretreatment.

### Corticosteroids increase catecholamine synthesis by a combination of mechanisms that affects its synthesis and storage

**Figure 2:**
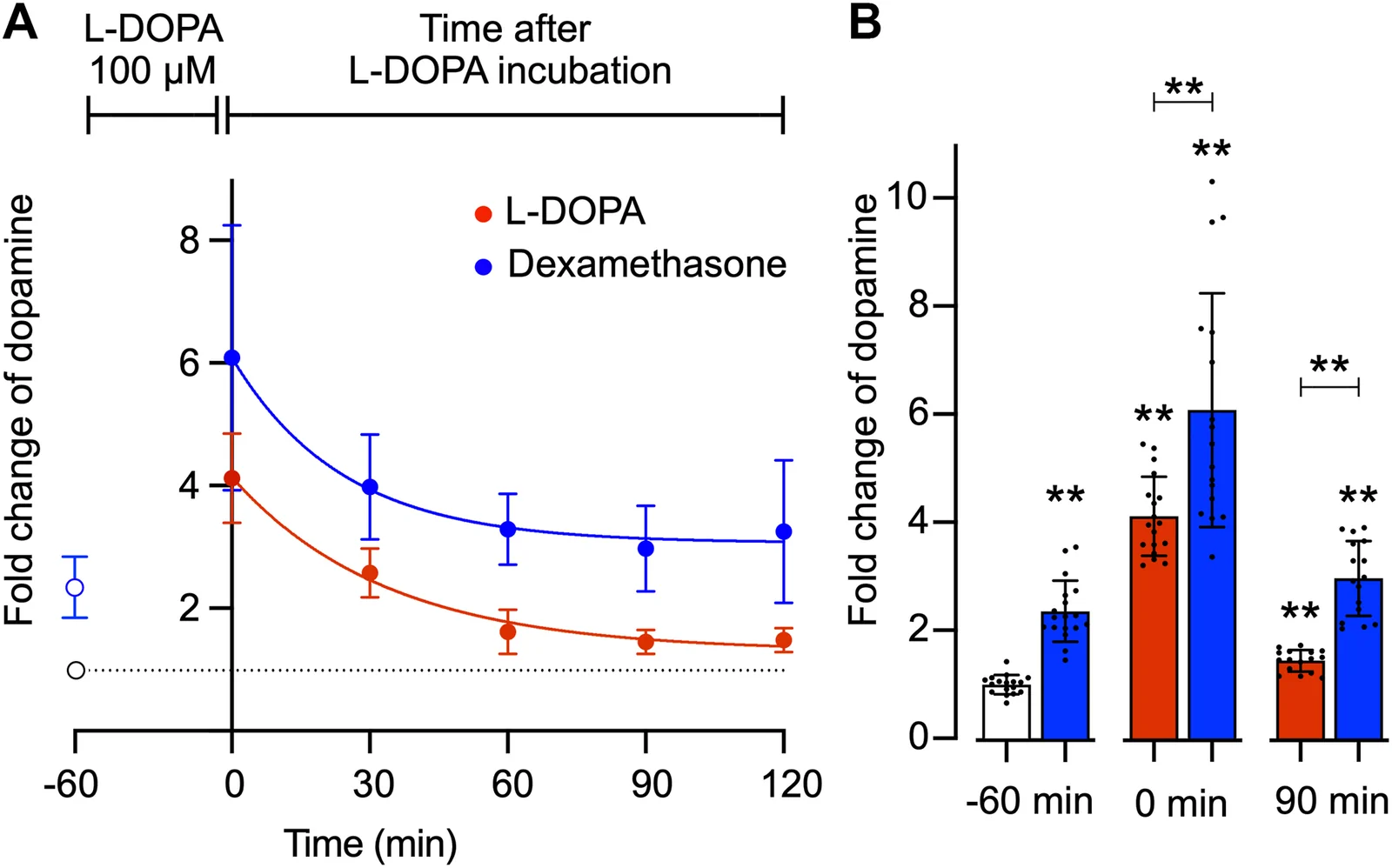
**Dexamethasone increases the dopamine uptake in a time dependent manner.***A,* DA content in cells treated for 48 h with DEX (100 nM). Then cells were treated with 100 μM L-DOPA for 60 min. The total DA was quantified using HPLC-ED. Data points represent the mean ± SD (n = 18 independent biological replicates per condition, collected across three independent experimental days). The *dashed horizontal line* denotes basal dopamine levels. The data for both conditions are best described by a single exponential decay curve, with a time constant of approximately 25 min. *B,* total DA content was measured immediately after L-DOPA incubation and after 90 min of washing out in cells untreated *(red bars)* and treated with DEX *(blue bars)*. Data (means ± SD of 18 independent biological replicates per condition, collected across three independent experimental days) were normalized to untreated cells. Statistical analyses were performed comparing basal condition indicated by hashtag (##*p* < 0.01) and between the basal condition (−60 min) in the different experimental groups at 0- and 90-min. Significance differences are indicated by *asterisk* ∗*p* < 0.05 and was assessed by two-way ANOVA followed by Tukey's *post hoc* multiple-comparisons test. DA, dopamine; DEX, dexamethasone.

To investigate whether DA uptake can be improved, we examined the time course of L-DOPA dependent dopamine accumulation in cells pretreated with 100 nM DEX for 48 h. DEX increased the basal DA content by approximately 2.2-fold compared with untreated cells (Fig. 2). At time 0, following L-DOPA incubation, DA levels were significantly higher in DEX-treated cells than in untreated cells, indicating an enhanced capacity for DA accumulation. Upon subsequent exposure to L-DOPA, DEX-treated cells displayed an additional rise in total DA levels, reaching ∼4.2- to ∼6.3-fold above baseline respectively. Following L-DOPA withdrawal, in both cases, intracellular DA content declined following a single-exponential decay with τ ∼25 min.

**Figure 3:**
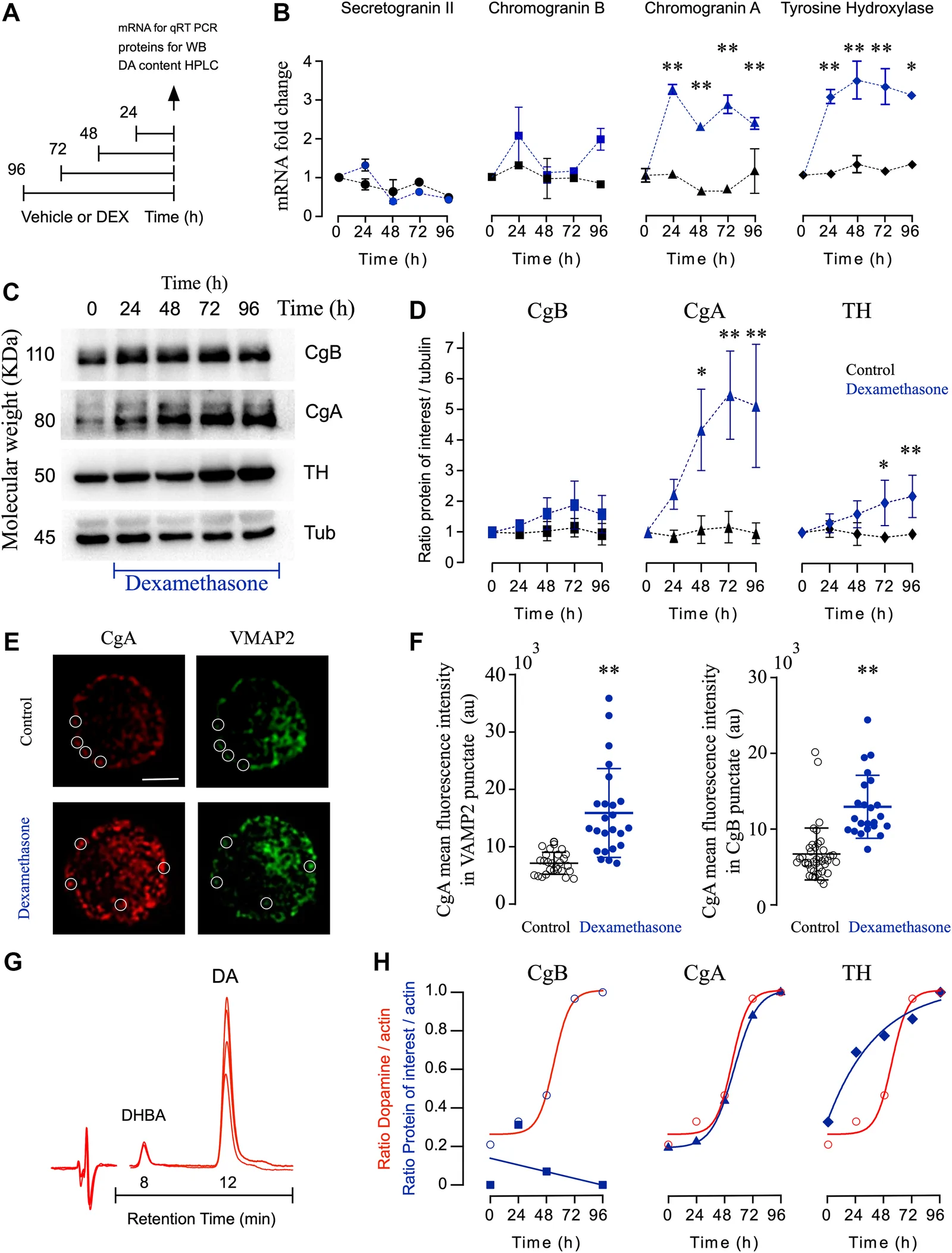
**Dexamethasone increases dopamine accumulation in parallel with CgA, but not TH or CgB.***A,* schematic illustration outlining the experimental design and workflow. *B,* time-course of mRNA expression levels for secretogranin II (*SCG2*), chromogranin B, chromogranin A, and *TH* in PC12 cells treated with DEX *(blue)* compared to untreated controls *(black)* at the indicated time points. Data represent mean ± SEM, normalized to *GAPDH* and *HPRT1* housekeeping, from three independent experiments (n = 3). *C,* representative Western blot images showing the expression levels of chromogranin B (CgB), chromogranin A (CgA), and TH at different time points (0, 24, 48, 72, and 96 h) of treatment with DEX e. Each lane contains equal amounts of protein (20 μg). Tubulin was used as an internal loading control (n = 3). *D,* time-course of CgB, CgA, and TH expression after treatment with DEX *(blue)* compared to untreated cells *(black)* at indicated times. Data are means ± SD normalized to tubulin replicates from three independent experiments, (n = 3) ∗*p* < 0.05 and ∗∗*p* < 0.01 (one-way ANOVA, with Dunnett's test). *E,* representative epifluorescence images showing CgA, VAMP2 and CgB immunoreactivity in control and DEX -treated PC12 cells. CgA *(red)* displays a punctate pattern distributed throughout the cytoplasm, consistent with localization in secretory vesicles. VAMP2 and CgB *(green)* display similar punctate distribution. Images are representative of 20 cells analyzed from the same culture. The scale bar represents 4 μm. *F,* quantitative analysis of CgA-VAMP2 and CgA-CgB positive puncta and mean fluorescence intensity in control and DEX -treated cells. Data are presented as individual punctate with mean ± SD. Statistical significance was determined using an unpaired Student’s *t* test. ∗∗*p* < 0.01. *G,* HPLC chromatograms showing representative measurements of DA content following incubation with DEX at different time points, as illustrated in *(A)*. *H,* quantification of DA content and expression levels of CgB, CgA, and TH, each normalized to actin. Temporal expression profiles were fitted using either a sigmoid or a single-exponential model, as appropriate. DA, dopamine; DEX, dexamethasone; TH, tyrosine hydroxylase.

Given that TH is the rate limiting step in catecholamine synthesis and chromogranins are the main protein components of the granular matrix, we evaluate the effects of DEX on gene and protein expression. To this end, we performed a time course experiment as illustrated in (Fig. 3*A*). We quantified mRNA levels of key catecholamine related genes at multiple time points (24, 48, 72 and 96 h). Consistent with previous reports (35, 36, 37, 38), DEX significantly increased the mRNA expression of *TH* and *CHGA*. As shown in Figure 3*B*, *TH* and *CHGA* mRNA levels were significantly upregulated, peaking at 24 h (*TH*: 2.9 ± 0.3-fold; *CHGA*: 3.5 ± 0.4-fold *versus* control, *p* < 0.001). After 24 h, expression levels plateaued and remained elevated through 72 h. In contrast, chromogranin B (*CHGB*) mRNA levels did not change significantly at any time point (*p* > 0.05). Similarly, no significant changes were observed in *SCG2* (secretogranin II) mRNA expression (see Fig. 3*B*). The increases in *TH* and *CHGA* transcript levels were followed by delayed but sustained rises in protein expression (Fig. 3, *C* and *D*). CgA protein levels increased nearly linearly from 24 to 72 h, reaching a plateau at ∼5.2 ± 0.5-fold above baseline. TH protein expression increased more gradually, reaching ∼2.1 ± 0.3-fold by 72 h. Although prior studies have reported DEX induced upregulation of *SLC18A2* mRNA expression (35), we were unable to reliably quantify its expression under our experimental conditions. Neither the tested qPCR primers nor the commercially available antibodies produced a sufficiently specific or detectable signal. These data are consistent with previous findings using the same PC12 cell line, in which SLC18A2 expression has proven difficult to detect (39, 40). Subsequently, to correlate TH and chromogranins expression with DA levels, we conducted time course experiments in DEX treated cells. We measured new set of TH, CgA, CgB expression levels (normalized to actin) and correlated with DA levels simultaneously on the same cells at each time point. As shown in Figure 3, *G* and *H*, DA accumulation closely paralleled the time course of CgA expression, rather than that of TH or, as initially expected, CgB.

To determine whether DEX increases CgA cargo specifically within secretory vesicles, we performed dual immunocytochemical labeling of CgA (red) together with two independent secretory vesicle markers, VAMP2 (green) and CgB (green), under control conditions and following incubation with 100 nM Dex for 48 h (Fig. 3*E*). Quantitative image analysis showed that DEX treatment did not significantly alter the number of CgA positive puncta per cell compared with control cells (*p* > 0.05). Quantification of CgA fluorescence intensity was restricted to puncta that co-localized with VAMP2 or CgB, ensuring that only structures corresponding to *bona fide* secretory vesicles were included in the analysis. Within this marker validated population, DEX treatment significantly increased CgA signal per vesicle relative to control (unpaired Student’s *t* test, *p* < 0.01) (Fig. 3*F*).

### L-DOPA increases vesicular content but does not alter the time course of secretion

**Figure 4:**
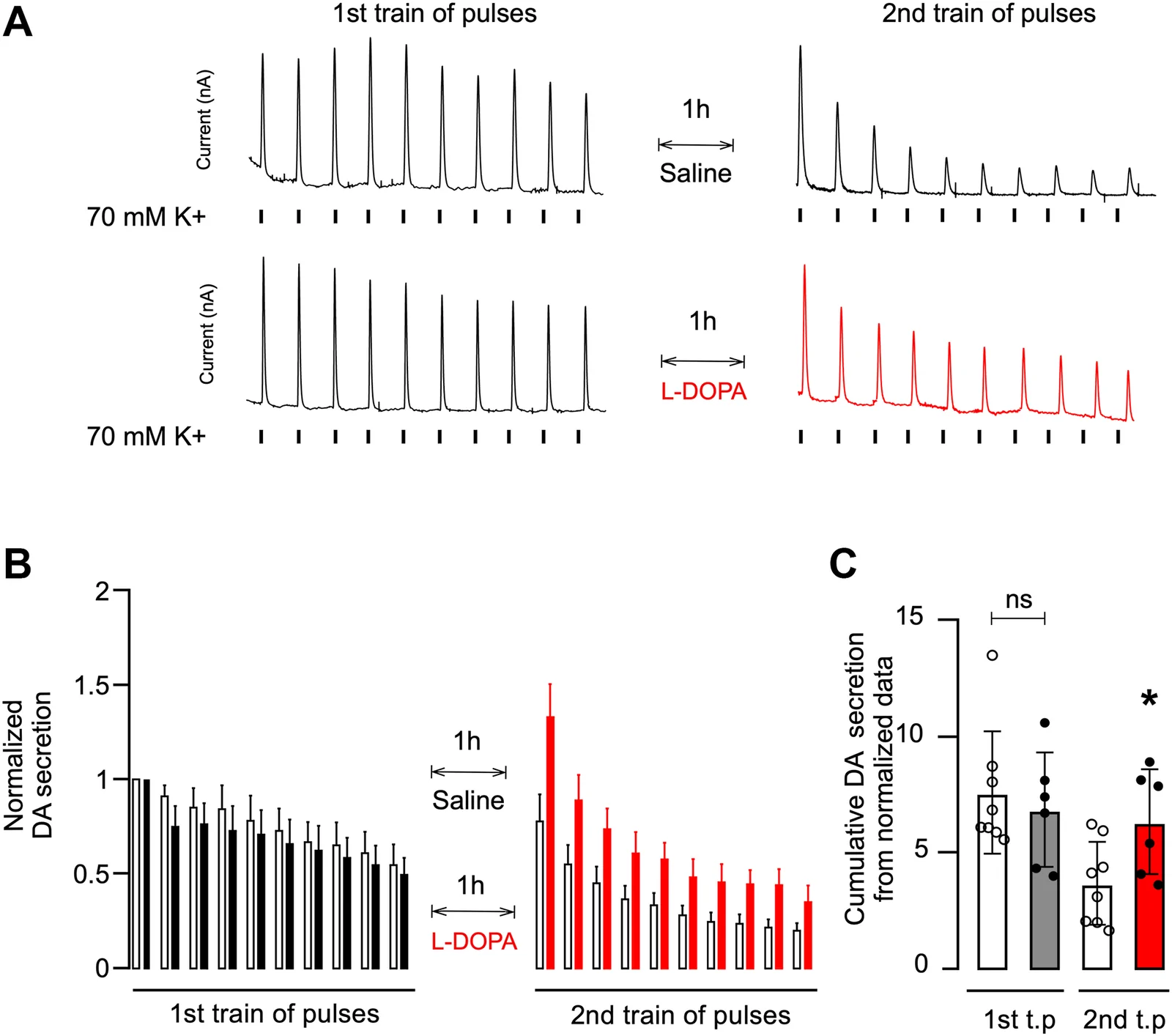
**L-DOPA increases vesicular content but does not alter the time course of secretion.***A,* Representative electrochemical recordings from perifused PC12 cells. Each current peak indicates dopamine secretion evoked by repetitive stimuli with 70 mM K^+^ in a Krebs-HEPES solution at 3-min intervals. The second trains of pulses were performed, separated by 1 h of perfusion in the absence *(white bars)* or presence *(red bars)* of 100 μM L-DOPA. *B,* time course of dopamine release from control and L-DOPA treated cells. In each experiment, the data were normalized to the area of the first secretory peak, to correct differences between the cellular batches. *C,* cumulative secretion obtained from untreated *(control; white bars)* and treated (L-DOPA; *black and red bars*) PC12. *Bars* represent the sum of mean areas ± SD; from eight independent subcultures (n = 8). ∗*p* < 0.05 (Student’s *t* test) in the first and second train of pulses.

To determine how L-DOPA–dependent DA reloading modulates the recovery of exocytotic responses after sustained stimulation, we used an on-line amperometry perfusion system coupled to electrochemical detection, which enables continuous real-time quantification of DA release from immobilized population of PC12 cells (see On-line amperometry in the *Methods* section for details). Cells were stimulated with a train of ten depolarizing pulses of 70 mM K^+^ in Krebs-HEPES solution at 3-min intervals (first train of pulses), designed to evoke exocytosis of the secretory pool of DA secretory vesicles (Fig. 4*A*). Each peak in the electrochemical trace corresponds to a discrete pulse of DA secretion induced by potassium. Following the first train of pulses, we stopped stimuli and cells were perfused with a saline buffer for 60 min (resting period), allowing new DA synthesis and intravesicular refilling of exhausted vesicles. A second identical train of 10 K^+^ pulses was then applied (second train of pulses), providing insight into the recovery of secretory capacity under baseline conditions. This approach enabled us to measure both the secretory dynamics and the capacity of PC12 cells to restore their releasable DA pools. The exogenous precursor enabled the synthesis of new DA, which could be taken up and stored in vesicles, including those previously depleted during the initial stimulation.

Representative electrochemical traces from both control and L-DOPA treated cells are shown in Figure 4*A*. In untreated cells, the amplitude of dopamine release during the second stimulation was markedly reduced compared to the first train of pulses, indicating a limited recovery of secretory function. The cumulative DA release during the second stimulation was reduced by 3.66 ± 0.42-fold relative to the first (n = 5, *p* < 0.01, paired *t* test; Fig. 4*C*). In contrast, cells exposed to L-DOPA during the recovery period maintained a robust secretory response. Their cumulative dopamine release remained stable, with a total output of 7.72 ± 0.68-fold over baseline levels, not significantly different from the first stimulation (n = 5, *p* > 0.05; Fig. 4*C*). These results demonstrate that newly synthesis significantly improves the replenishment of releasable DA stores, likely through enhanced reuptake and vesicle refilling mechanisms.

### Glucocorticoids increase dopamine release and reduce the decaying of repetitive secretion

**Figure 5:**
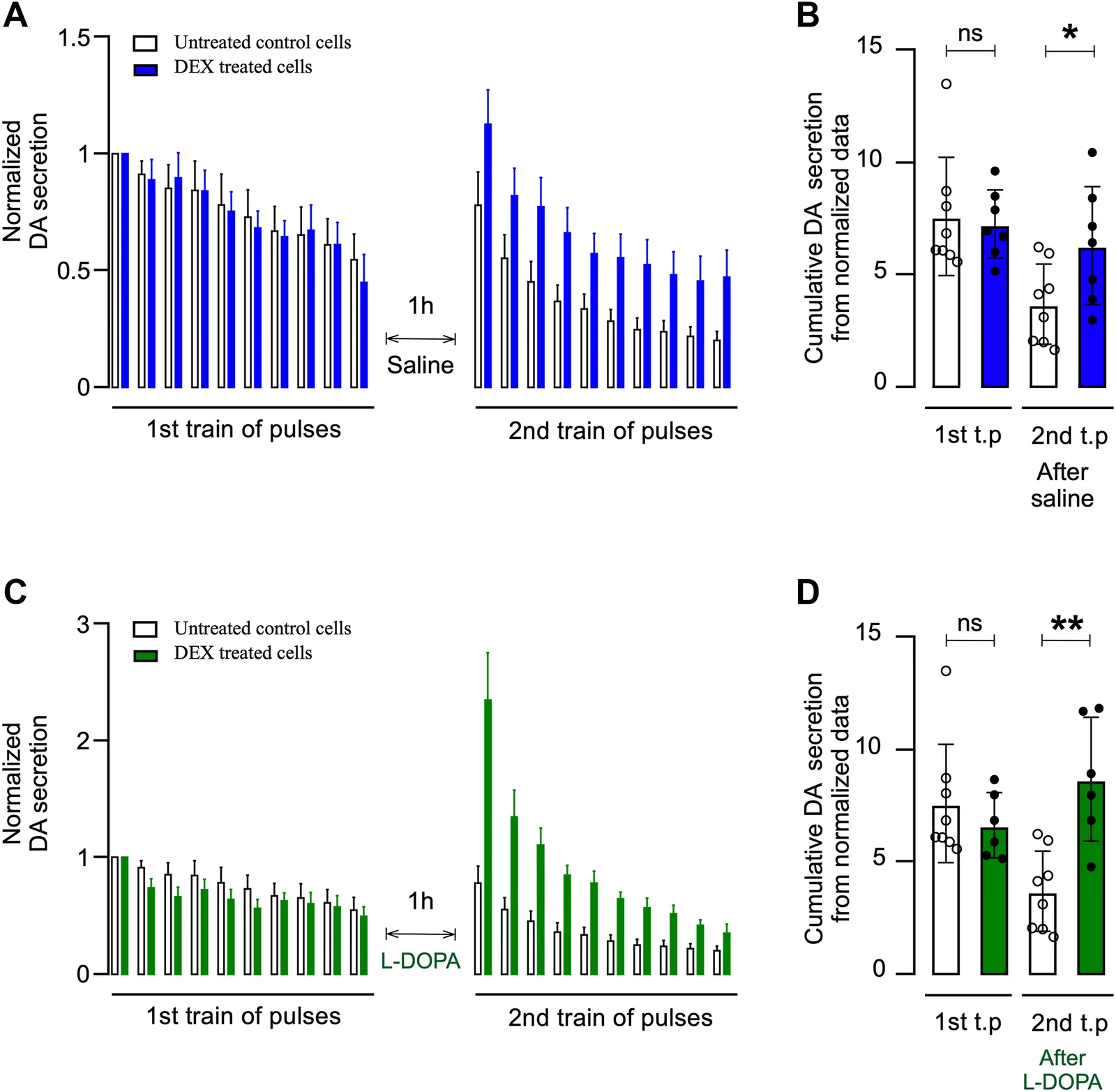
**Differences in the secretory time-course between DEX and L-DOPA.***A,* time course of dopamine release from control cells (8 independent subcultures; n = 8; *white bars*) and cells previously treated with 100 nM DEX (6 independent subcultures; n = 6; *blue bars*) for 48 h. Stimuli were stopped for 60 min between the two trains of pulses. *C,* graph represents same experimental conditions as A, but treated cells were perfused with 100 μM L-DOPA for 1 h, between both trains of pulses. Data were normalized to the area of the first secretory peak, to correct differences between the cell batches. *Band D,* cumulative secretion obtained from untreated *(white bars)* and treated *(blue and green bars)* cells. *Bars* represent the integration of peak areas (means ± SD). ∗*p* < 0.05, ∗∗*p* < 0.01 (Student’s *t* test) in the first and second train of pulses. DEX, dexamethasone.

Secretion experiments using the same on-line amperometry perfusion system coupled to real-time electrochemical detection, were performed in cells treated with DEX (100 nM) for 48 h. Following the first train of K^+^ pulses cells underwent a 60 min resting period followed by the second train of pulses. During the second train of pulses, DEX treated cells displayed a significantly enhanced dopamine secretion compared to untreated controls (Fig. 5*A*). The cumulative DA release during the second stimulation increased by 6.66 ± 0.51-fold over untreated control cells (*n* = 6, *p* < 0.01 *versus* control; Fig. 5*B*), despite the absence of exogenous L-DOPA. This response mimicked the secretion dynamics observed in L-DOPA treated cells (Fig. 4), consistent with similar secretion decay kinetics (τ ≈ 25 min in both cases), suggesting that glucocorticoid treatment enhances endogenous DA synthesis and vesicular refilling. When DEX treated cells were additionally perfused with L-DOPA (100 μM) during the recovery period (Fig. 5*C*), the dopamine release was significantly greater. The cumulative secretion reached 9.11 ± 0.84-fold over untreated control cells (*n* = 6, *p* < 0.001; Fig. 5*D*). Notably, the enhancement was particularly prominent during the initial pulses of the second train, with a secretion decay constant (τ) of approximately 25 min (Fig. 5*C*), indicating that the effect is primarily exerted on the first pool of DA secretory vesicles. In contrast, untreated PC12 cells showed a marked decline in secretory capacity over time, with cumulative DA release during the second stimulation decreasing by ∼55% compared to the first (3.66 ± 0.42-fold; *p* < 0.01), highlighting the limited ability of the cells to recover and sustain secretion under basal conditions.

Together, these results indicate that DEX enhances vesicular DA storage and recycling, mimicking and synergizing with the effects of L-DOPA. The combination of both treatments sustains DA secretion during prolonged stimulation and preferentially enhances release from the most responsive vesicle pools, as evidenced by the early high amplitude secretion peaks.

### Dexamethasone and L-DOPA synergistically enhance dopamine quantal release through increased vesicle storage capacity

**Figure 6:**
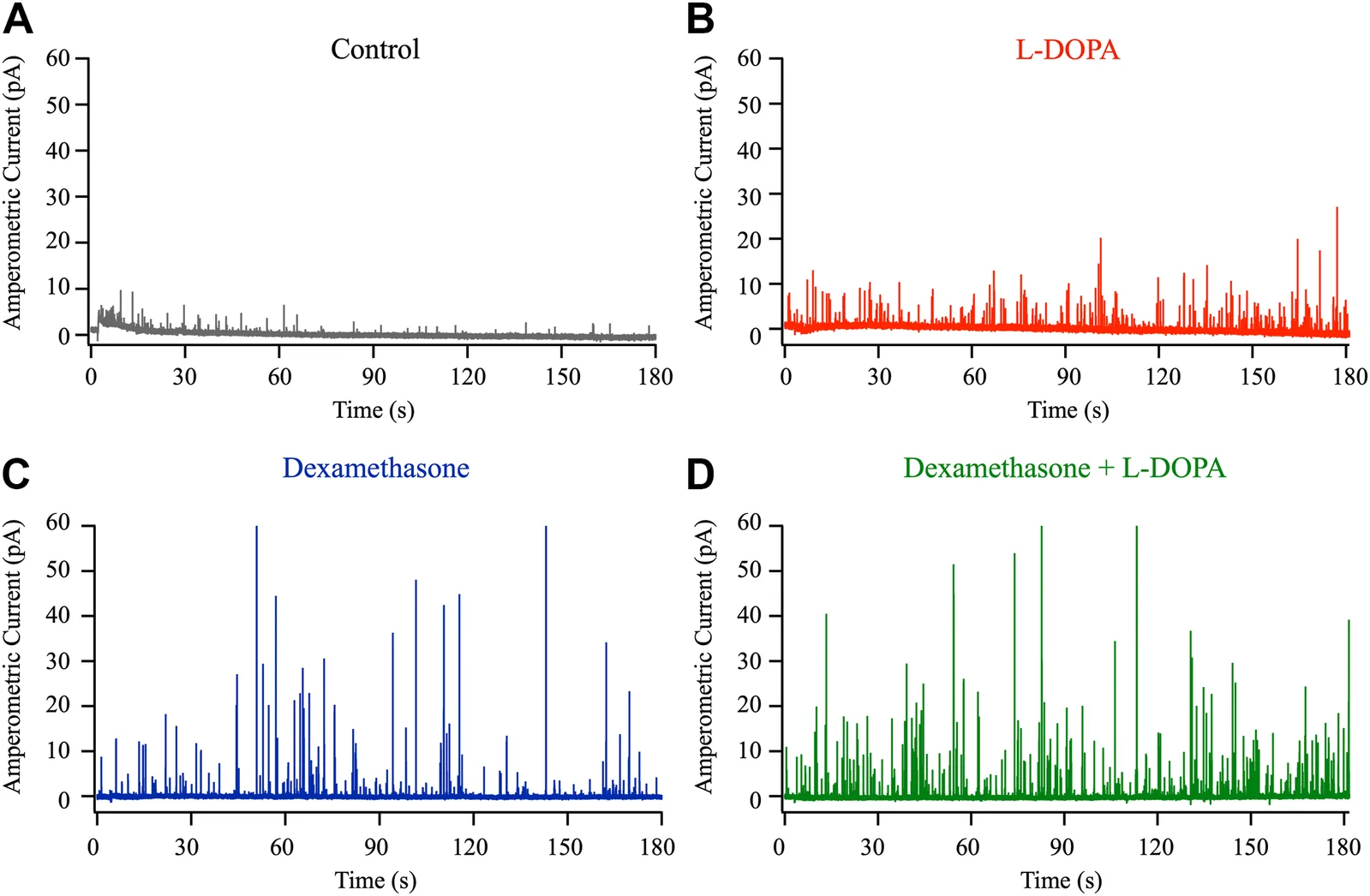
**Representative amperometric recordings of dopamine release from PC12 cells under different pharmacological conditions.** Amperometric currents were recorded for 180 s using carbon-fiber microelectrodes to monitor single-vesicle exocytotic events elicited after barium injection. *A,* control cells displayed low basal secretory activity and low-amplitude spikes. *B,* L-DOPA treated cells exhibited an increased frequency of release events, consistent with enhanced vesicular catecholamine loading. *C,* dexamethasone-treated cells showed a pronounced elevation in both spike frequency and amplitude, indicating potentiation of stimulus-evoked exocytosis. *D,* combined DEX + L-DOPA treatment produced robust secretory activity comparable to DEX alone, characterized by numerous high-amplitude spikes. Traces illustrate treatment dependent modulation of vesicular release dynamics. DEX, dexamethasone.

The capacity of secretory vesicles to accumulate and release DA is critically dependent on the presence of intravesicular proteins such as CgA, which serve as matrix components that facilitate monoamine condensation. Since glucocorticoids upregulate CgA expression in PC12 cells (Fig. 3) and CgA has been described as a key contributor of secretory vesicle biogenesis (41), we hypothesized that DEX treatment would promote the formation of newly synthesized vesicles enriched in CgA, thereby enhancing their DA storage capacity. To test this hypothesis, we examined frequency of exocytosis events and the kinetics and quantal properties of individual exocytotic events in PC12 cells using high resolution single-cell amperometry. Exocytosis was triggered by a 5-s pressure ejection of 5 mM BaCl_2_, a non-receptor mediated secretagogue that induces Ca^2+^ influx *via* voltage-gated calcium channels (42). This stimulation protocol elicited a consistent, low-frequency secretory response, enabling reliable detection and analysis of discrete amperometric spikes (Fig. 6).

Under control conditions, PC12 cells exhibited a mean exocytotic frequency of ∼0.8 Hz, with a quantal size (Q) of 20.4 ± 12.0 fC and a peak current (Imax) of 3.5 ± 1.8 pA. These parameters remained stable throughout the stimulation period, indicating uniform vesicle release (Table 1). Dexamethasone treatment (100 nM, 48 h) enhanced exocytosis, raising the events frequency from ∼0.8 Hz to ∼1.1 Hz, (unpaired Student´s *t* test; *p* < 0.01), after secretagogue stimulation, reflecting a substantial enhancement in release frequency. Dexamethasone also significantly increased quantal size (84.4 ± 56 fC, *p* < 0.01 *versus* control) and peak current (11.1 ± 3.6 pA, *p* < 0.01), corresponding to 4.14-fold and 3.17-fold increases, respectively. While the half-width duration (t_1_/_2_) of amperometric spikes remained unchanged (5.8 ± 1.9 ms, *p* > 0.05), the slope of the ascending phase (m) a proxy for release kinetics increased significantly (4.6 ± 1.2 pA/ms, *p* = 0.052), indicating a positive correlation with maximum peak current. L-DOPA treatment alone (100 μM, 1 h preincubation) also potentiated secretion, though to a lesser extent. Quantal size reached to 56.9 ± 7.0 fC (*p* < 0.01) and peak current to 7.0 ± 1.6 pA (*p* > 0.05), representing 2.79-fold and 2.00-fold increases over control, respectively. However, L-DOPA had minimal effects on spike duration (t_1/2_ = 6.5 ± 1.5 *versus* 5.5 ± 1.3 ms) and slope (*m* = 2.9 ± 1.1 *versus* 1.9 ± 1.6 pA/ms), suggesting that its primary effect was to enhance vesicle filling rather than alter release kinetics. Strikingly, the combination of DEX and L-DOPA produced a synergistic enhancement of exocytosis. Quantal size reached 151.9 ± 71.0 fC (*p* < 0.01) and peak current increased to 14.9 ± 5.5 pA (*p* < 0.01), corresponding to 7.45-fold and 4.26-fold increases over control, respectively. This was accompanied by a modest but significant increase in t_1_/_2_ (7.9 ± 1.8 ms, *p* = 0.052) and a further rise in slope (4.9 ± 1.9 pA/ms, *p* < 0.05), consistent with a larger and more forceful release of DA per vesicle (Table 1).

**Table 1:** Amperometric spike characteristics obtained in PC12 cells treated with L-DOPA in the presence or absence of dexamethasone

Experiment	*I*_*max*_ (pA)	*p* value	Q (fC)	*p* value	t1/2 (ms)	*p* value	m (pA/ms)	*p* value	N (cells)	n (spikes)
Control	3.5 ± 1.8	*p* = 0.0260 Mann–Whitney	20.4 ± 12	*p* = 0.0022 Mann–Whitney	5.5 ± 1.3	*p* = 0.3095 Mann–Whitney	1.9 ± 1.6	*p* = 0.0931 Mann–Whitney	6	217
L-DOPA	7.0 ± 1.6	*p* = 0.0780 Holm–adjusted p	56.9 ± 7^a^	*p* = 0.0088 Holm–adjusted p	6.5 ± 1.5	*p* = 0.3095 Holm–adjusted p	2.9 ± 1.1	*p* = 0.1862 Holm–adjusted p	6	604
Fold change	2.00		2.79		1.18		1.52			
Dex	11.1 ± 3.6^a^	*p* = 0.0022 Mann–Whitney	84.4 ± 56^a^	*p* = 0.0022 Mann–Whitney	5.8 ± 1.9	*p* = 0.8182 Mann–Whitey	4.6 ± 1.2^b^	*p* = 0.0152 Mann–Whitney	6	594
		*p* = 0.0088 Holm–adjusted p		*p* = 0.0088 Holm–adjusted p		*p* = 0.8182 Holm–adjusted p		*p* = 0.0304 Holm–adjusted p		
Fold change	3.17		4.14		1.05		2.42			
Dex + L-DOPA	14.9 ± 5.5^a^	*p* = 0.0022 Mann–Whitney	151.9 ± 71^a^	*p* = 0.0022 Mann–Whitney	7.9^b^ ± 1.8	*p* = 0.0411 Mann–Whitney	4.9 ± 1.9^b^	*p* = 0.0260 Mann–Whitney	6	495
		*p* = 0.0088 Holm–adjusted p		*p* = 0.0088 Holm–adjusted p		*p* = 0.0520 Holm–adjusted p		*p* = 0.0520 Holm–adjusted p		
Fold change	4.26		7.45		1.44		2.58			

Data from cells recorded from the same culture, on the same day. Recordings were performed by alternating between cells treated with L-DOPA and control cells to minimize temporal recording bias. Statistical analyses were conducted at the cell level (n = 6 cells per group). For each cell provides at least 30 spikes, which pass the criteria specified in Material and Methods section. Data are expressed as means ± SD (units indicated in parentheses). Group comparisons were performed using the Mann–Whitney *U* test. Because four spike parameters (Imax, Q, t1/2, and m) were analyzed per comparison, *p*-values were corrected for multiple testing using the Holm–Bonferroni method. Raw *p* values and adjusted *p*-values are reported in Table 1. Statistical significance after correction is indicated as.

a *p* < 0.01.

b *p* < 0.05.

**Figure 7:**
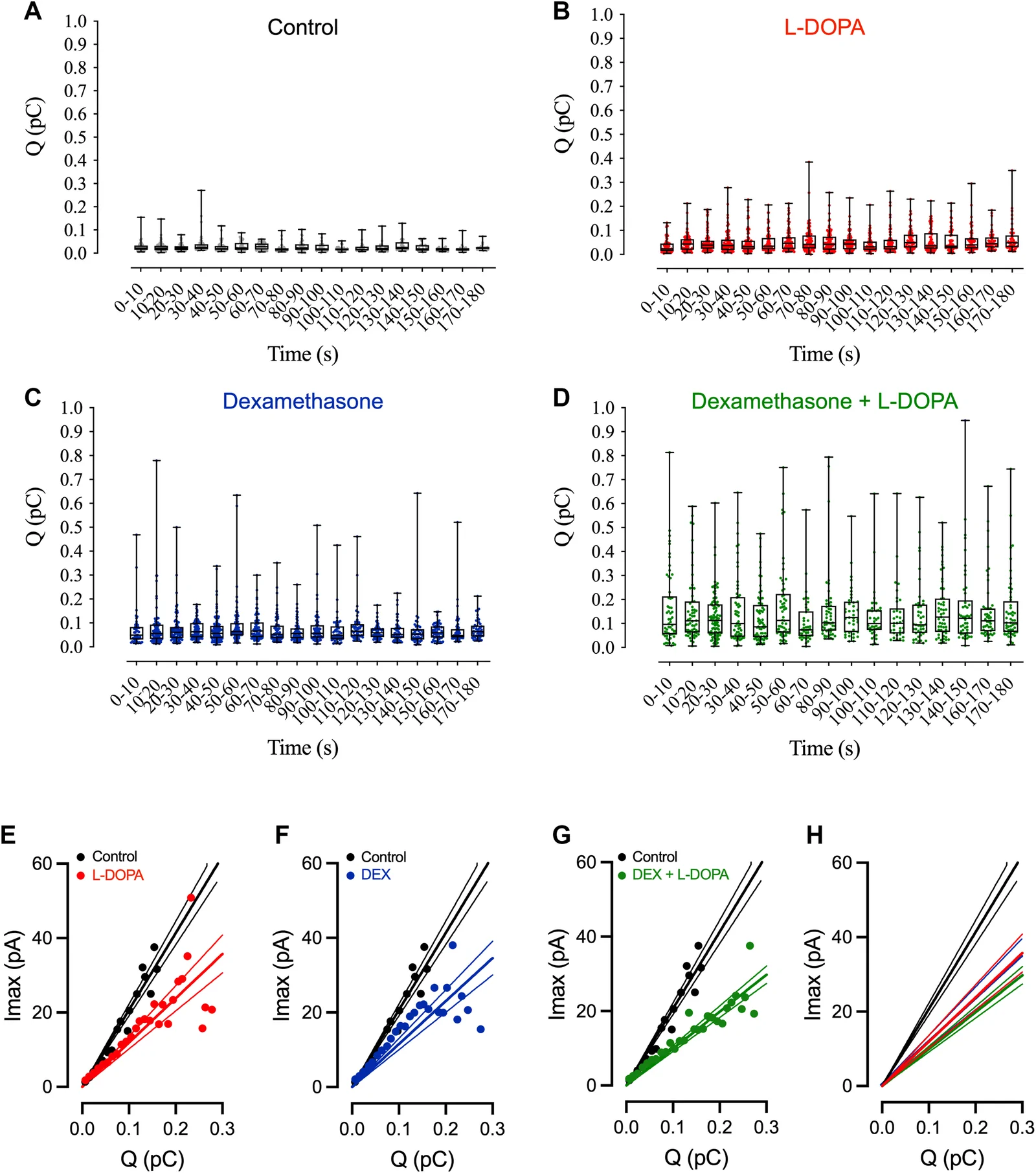
**Analysis of quantal size over time to assess pre-existing and newly formed vesicles.** Box-and-whisker plots show the distribution of quantal size (Q, pC) for exocytotic events recorded across successive 10-s time segments from the start of the amperometric recording. Data are shown for *(A)* Control, *(B)* L-DOPA, *(C)* Dexamethasone, and *(D)* Dexamethasone + L-DOPA conditions. This temporal segmentation was used to test, whether vesicles released earlier *versus* later in the recording differed in their storage capacity. Relationship between catecholamine content of vesicles *(Q)* and the maximal concentration reaching the carbon fiber electrode (Imax) under the influence of L-DOPA overload. Spikes from different experimental conditions from Figure 6 were combined, sorted, and divided into 30 intervals based on charge (increments of 0.01 pC). The spikes were then categorized into control *(black) dots*, L-DOPA *(red)*; *(E)*, DEX; *(blue)*; *(F)*, and Dex + L-DOPA *(green) (G)*; different experimental conditions, and their Imax values were plotted *(H)*. Notably, spikes from the control group exhibited larger Imax values compared to other conditions at similar quantal sizes. *Lines* represent simple linear regression and 95% confidence intervals (pA/pQ). Control; slope: 206.7 ± 7.4 (R^2^ = 0.93), L-DOPA; slope 119.1 ± 8.2 (R^2^ = 0.64); DEX; slope 115.3 ± 7.3 (R^2^ = 0.62) and DEX + L-DOPA slope; 99.2 ± 3.8 (R^2^ = 0.85). Pairwise comparisons of regression slopes were adjusted for multiple testing using the Benjamini–Hochberg false discovery rate procedure. Differences between Control *versus* L-DOPA raw *p* = 0.0006; p FDR (BH) = 0.0012; Control *versus* DEX raw *p* = 0.0004; p FDR (BH) = 0.0012; Control *versus* DEX + L-DOPA raw *p* = 0.0001; p FDR (BH) = 0.0006; L-DOPA *versus* DEX raw *p* = 0.8927; p FDR (BH) = 0.8927; L-DOPA *versus* DEX + L-DOPA raw *p* = 0.3387; p FDR (BH) = 0.5081; DEX *versus* DEX + L-DOPA raw *p* = 0.4347; p FDR (BH) = 0.5216. All treated conditions remained significant after FDR correction (adjusted *p* < 0.01). DEX, dexamethasone.

To further address the possibility of coexisting pre-existing and newly formed vesicle populations and the temporal preference for been released, we performed an additional amperometric analysis in which exocytotic events were grouped according to their time of occurrence during the recording, following the procedure described by (20). To determine whether the observed changes in quantal size reflect the recruitment of different vesicle populations, we categorized exocytotic events based on their timing during the recording. For each time segment (10 s), we quantified the quantal size and compared the resulting distributions across the entire recording window (180 s) (Fig. 7). If pre-existing and newly formed vesicles differed in their quantal size or the temporal preference for exocytosis, this approach would reveal subpopulations with distinct amount of DA released. However, no significant differences were detected in any experimental condition across time segments (Mann–Whitney *U* test with Bonferroni correction, ns, *p* > 0.05), indicating that the vesicles released during stimulation constitute a functionally homogeneous population with respect to quantal size. To further dissect the kinetic characteristics of exocytosis, we performed a comparative analysis of peak current (Imax) distributions across spikes of equivalent DA content. Amperometric spikes from all experimental conditions were pooled, sorted by quantal charge and binned into 30 discrete intervals of 0.01 pC each. Within each bin, spikes were assigned to their respective treatment groups: control (black), L-DOPA (red), DEX (blue) and DEX plus L-DOPA (green); the mean Imax was calculated and plotted (Fig. 7). This approach enabled a direct comparison of release kinetics independent of vesicle size, revealing treatment dependent shifts in Imax profiles across matched quantal ranges (43). As shown in Figure 7, control cells (Fig. 7*E*, red closed circles) exhibited higher Imax per unit charge (206 pA/pC) compared to DEX treated cells (Fig. 7*F*, blue closed circles) and those treated with both DEX and L-DOPA (Fig. 7*G*, green closed circles), which showed reduced values (∼100 pA/pC). This reduction in normalized peak current suggests that increased vesicular DA content, likely reflecting a shift in the ratio of free to protein bound transmitter, modulates the kinetics of release, slowing the rate of expulsion despite enhanced vesicle filling.

## Discussion

This study shows that DEX coordinately remodels vesicular composition and improve DA secretion in PC12 cells. We used this cell line as a mechanistically tractable model to study how prolonged exposure to glucocorticoids remodels functional properties of secretory vesicles. In the intact adrenal gland, chromaffin cells are continuously exposed to glucocorticoids released from the surrounding adrenal cortex through the corticomedullary portal circulation. However, glucocorticoid exposure is highly dynamic and is shaped by circadian and pulsatile glucocorticoid oscillations, which are further amplified during stress (44). These fluctuations are thought to fine tune the catecholaminergic phenotype by regulating multiple aspects of chromaffin cell function, including catecholamine biosynthesis, secretory granule maturation, and secretion (29). Although the present study employed sustained DEX exposure to investigate the underlying cellular mechanisms, our findings raise the possibility that physiological pulsatile glucocorticoid oscillations may also contribute to the temporal coordination of *CHGA* dependent intravesicular matrix assembly with dopamine loading, thereby optimizing vesicular storage capacity and secretory function (45). The proliferative capacity of PC12 cells, together with their continuous production of secretory vesicles during sustained corticosteroid treatment, provides a distinct experimental advantage for analyzing transcription dependent modifications in vesicular cargo loading. Using this system, we were able to directly assess the influence of glucocorticoid induced upregulation of vesicular components, including CgA, on intravesicular amine accumulation and quantal release characteristics.

Glucocorticoid signaling in PC12 cells is known to be highly pleiotropic, encompassing broad transcriptional programs, protein phosphorylation, cytoskeletal remodeling and changes in cellular phenotype (32, 46). Our data fit within this complex framework by showing that sustained DEX incubation, which is a well-established differentiation protocol (14, 32), increases the expression of two key catecholamine related proteins (TH and CgA), expands intravesicular storage capacity and, especially when combined with L-DOPA, produces larger quantal release events and more persistent secretion. Although this experimental paradigm does not reproduce the physiological circadian fluctuations of endogenous corticosteroids, its effect on TH expression in adrenal medulla is consistent with that observed in animals exposed to stress (35). Taken together, expression analysis, population level secretion measurements and single vesicle kinetic data support a coherent mechanism whereby glucocorticoid signaling drives the formation of DA secretory vesicles enriched in CgA, thereby enhancing vesicular DA storage capacity.

### Molecular drivers of enhanced vesicular storage

Dexamethasone increases mRNA level of *CHGA* and *TH* and induced robust upregulation of CgA (38) and TH (35) (Fig. 3). Although TH elevation increases DA synthesis, the temporal covariation we observe between CgA expression and DA accumulation implicates matrix remodeling as the dominant determinant of sustained vesicular storage in PC12 cells. Consistent with this interpretation, immunofluorescence analysis demonstrated that DEX did not alter the number of CgA-positive puncta and no obvious differences in the overall cellular distribution were observed between control and DEX treated cells (data none shown). Additionally, significantly increased the fluorescence intensity per puncta, suggesting that DEX enhances the amount of CgA per secretory vesicle. Increased CgA plausibly augments intravesicular binding sites and matrix volume (18, 47, 48), permitting condensation of greater DA amounts. This matrix-based mechanism explains why DEX elevates basal DA stores (Fig. 2 and Table 1) and sustains intravesicular DA levels over time.

These results are consistent with the established role of chromogranins in forming a dense proteinaceous matrix that complexes catecholamines with chromogranins *via* electrostatic interactions (49). CgA depletion reduces vesicular storage and quantal size in chromaffin cells (8, 50), supporting our mechanistic interpretation. Importantly, under conditions of CgA deficiency, compensatory mechanisms are activated that lead to increased CgB expression (8). In contrast, experimental manipulations that reduce the expression of either CgA or CgB do not affect SgII expression, as shown in Figure 3*B* and in previous reports (8, 10, 50). Conversely, CgA overexpression does not elicit reciprocal compensatory changes in CgB expression (18) a finding that is also confirmed in the present work. This mechanistic emphasis on matrix capacity also aligns with classic models showing that vesicular protein content determines the osmotic and buffering properties of LDCVs (51).

### Kinetics of DA uptake and the role of its precursor supply

L-DOPA produces a rapid, transient rise in intracellular DA with a ∼30 min time constant, consistent with rapid synthesis followed by vesicular sequestration and cytosolic clearance (Fig. 1). This temporal profile aligns with established kinetics measured in PC12 and dopaminergic neurons (33, 52). Reserpine abolishes this accumulation, confirming SLC18A2-dependent uptake (53), while pargyline increases cytosolic DA (33, 34) but does not change long-term storage, underscoring that vesicular uptake, not simply cytosolic availability, limits sustained retention. Likewise, pargyline increases cytosolic DA but does not alter long-term storage, consistent with the principle that SLC18A2 transport capacity, not cytosolic substrate levels alone, sets storage limits (54). Dexamethasone preconditioning amplifies both the amplitude and persistence of L-DOPA driven accumulation, (Fig. 2), indicating that glucocorticoid dependent changes increase the effective capacity and efficiency of SLC18A2 mediated sequestration by providing an expanded intravesicular matrix that stabilizes sequestered transmitter.

### Functional consequences for vesicle recycling and population secretion

On-line secretion assays reveal that vesicular refilling is a rate limiting step for maintaining secretory capacity: L-DOPA supplied during the recovery interval preserves the second train response, demonstrating accelerated refilling (Fig. 4). Dexamethasone pretreatment produces a similarly enhanced recovery even without exogenous L-DOPA (Fig. 5), consistent with increased endogenous synthesis plus improved storage efficiency. The combination of DEX and L-DOPA produces the largest cumulative output, with early high amplitude peaks reflecting preferential enhancement of secretory vesicle pool. Real-time electrochemistry experiments performed in population of cells validate that the increase of expression level of TH and CgA and the quantal changes detected at single vesicle level, suggesting a superior ability to sustain evoked secretion under repetitive stimulation.

### Single vesicle kinetics and mechanistic interpretation

Single cell amperometry shows substantially larger quantal sizes and higher Imax after DEX treatment, with only modest or no change in spike duration. In addition, DEX produced a small but consistent increase in the frequency of exocytotic events. The selective enhancement of quantal parameters, in the absence of major alterations in spike duration, suggests that DEX primarily augments vesicular catecholamine content rather than markedly altering fusion pore dynamics. This interpretation is further supported by our molecular data demonstrating coordinated upregulation of the vesicular storage machinery, including chromogranins and dopamine biosynthetic enzymes, suggesting that glucocorticoids expand the functional storage capacity of secretory vesicles. In contrast, L-DOPA primarily increases cytosolic dopamine availability, thereby providing additional substrate for vesicular uptake without directly modifying vesicle architecture. Accordingly, the combined treatment produced the largest quantal size, indicating that the expanded storage capacity generated by DEX can be more efficiently utilized when dopamine precursor availability is no longer limiting. Notably, this additive effect was most evident for quantal size, whereas other amperometric parameters, particularly Imax, remained closer to DEX alone. This distinction is mechanistically important because quantal size reflects the total amount of transmitter released per vesicle, whereas Imax is also determined by fusion pore expansion, release kinetics, and the rate of transmitter efflux. Consequently, increasing vesicular content does not necessarily produce a proportional increase in peak current. The steeper ascending slope in DEX conditions indicates that more neurotransmitter is available for rapid expulsion when fusion occurs. Yet, when Imax is normalized to charge (pA/pC), treated cells show reduced normalized peak currents, indicating slower release per unit transmitter in heavily filled vesicles. Together with the prolonged half-width observed under the combined treatment, this pattern is most parsimoniously explained by an increased fraction of matrix-bound DA. Matrix associated transmitter contributes to larger Q but is released more slowly than freely diffusible DA, thereby altering the temporal profile of release while increasing total output while subtly modifying the temporal profile of exocytosis (8, 10, 50).

### Physiological and translational implications

Glucocorticoids thus emerge as potent modulators of neurosecretory response that act on both supply (TH induction) and storage (CgA-dependent matrix expansion). In neuroendocrine and stress contexts, such coordinated regulation would increase the magnitude and resilience of catecholaminergic signaling when sustained output is required. Clinically, these mechanisms suggest routes to modulate vesicular storage capacity pharmacologically: enhancing matrix components or substrate supply could augment transmitter output in conditions of deficient dopaminergic transmission, while excessive matrix expansion might contribute to maladaptive hypersecretion in stress related dysfunctions. Although DEX enhanced vesicular dopamine storage and increased quantal release in our PC12 cell model, the *in vivo* consequences of chronic glucocorticoid exposure are considerably more complex. Prolonged elevations of glucocorticoids, such as those occurring during sustained stress, hypercortisolism or long-term therapeutic corticosteroid treatment, affect multiple organs and systems, including aminergic pathways at several levels, such as neurotransmitter synthesis, vesicular packaging, release probability, receptor sensitivity and network level plasticity. Our findings therefore do not attempt to recapitulate the full physiological or pathological spectrum of chronic glucocorticoid exposure. Rather, they define a specific cellular mechanism: sustained glucocorticoid signaling can increase vesicular storage capacity and augment quantal dopamine release through coordinated regulation of vesicular components. Although this mechanism is clearly demonstrated in neuroendocrine cells, extrapolation of these findings to central dopaminergic neurons should be made with caution, given the distinct molecular and functional characteristics of these cell types. In this context, enhanced vesicular filling represents one component of a broader, multilevel response of aminergic systems to prolonged glucocorticoid exposure. In conclusion, DEX reprograms vesicular storage and release in PC12 cells through coordinated induction of synthetic enzymes and matrix proteins, producing larger, more resilient quantal events and superior recovery of secretory capacity. When combined with increased precursor availability, these changes synergistically maximize vesicular DA output. These results define a mechanistic framework in which glucocorticoid dependent matrix expansion and substrate supply jointly regulate both the magnitude and temporal dynamics of catecholamine secretion, with implications for adaptive and maladaptive modulation of aminergic systems.

## Experimental procedure

### Cell line culture

PC12 cells (CRL-1721; RRID: CVCL_0481) were obtained from (American Type Culture Collection. The cell line was provided by the supplier as contamination free and was routinely handled following sterile techniques. PC12 cells were maintained in suspension culture in RPMI 1640 medium (Sigma-Aldrich, R6504) supplemented with 5% fetal bovine serum (Biowest SAS, S1810), 5% horse serum (Biowest SAS, S0900), and penicillin–streptomycin solution (Gibco, 15070063). Cells were cultured at 37 °C in a humidified atmosphere with 5% CO_2_ and passaged every 2 to 3 days using a trypsin-EDTA free protocol. Only non-adherent cells were collected for passaging and used for experiments. The maximum number of passages carried out was 10 to avoid changes in cell function.

### Dopamine determination by HPLC-ED

The day prior to the experiment, PC12 cells (3 × 10^5^) were seeded in poly-D-lysine (Sigma-Aldrich, P1024) treated 24-well plate in RPMI 1640 supplemented with sera. To prepare for the different experimental conditions, cells were incubated with sera and antibiotics-free RMPI 1640 medium containing 100 μM L-DOPA (Sigma-Aldrich, D9628), 10 μM pargyline (Sigma-Aldrich, P8013) and 10 μM reserpine (Sigma-Aldrich, R0875) during different set times at 37 °C. Additionally, PC12 cells were pre-incubated in supplemented RPMI medium containing 100 nM DEX (DEX) (Sigma-Aldrich, D4902) for 48 h before undergoing a 60-min treatment with L-DOPA. The cells were then washed twice with Krebs-HEPES buffer (K-H; in mM) NaCl (140), KCl (5.9), MgCl_2_ (1.2), CaCl_2_ (2), HEPES (Sigma-Aldrich, H3375) (10), and glucose (11) at pH 7.4) and placed back into serum and antibiotics free RPMI-1640 medium for 30, 60, 90 or 120 min.

At the specified time points, the culture medium was removed, and the cells were triturated in ice-cold TENT lysis solution consisted of Tris HCl (50 mM), EDTA (5 mM), NaCl (150 mM) and Triton X-100 (1%) (Sigma-Aldrich, T9284) pH adjusted to ∼8.0. The homogenates were then centrifuged at 12,000×*g* for 10 min, and the supernatant was separated from the pellet and mixed a solution containing perchloric acid and 3,4-dihydroxybenzylamine (DHBA) (Sigma-Aldrich, 858781) as internal standard to achieve a final concentration of 0.05 N and 200 nM respectively. An aliquot of 50 μl was injected into HPLC LC-40 (Shimadzu) coupled to an electrochemical detector LC-4B (BioAnalytical Sciences).

### Gene expression analysis

Total RNA was extracted using RNAzol RT (Sigma-Aldrich) according to manufacturer's instructions and stored at −80 °C. RNA was quantified using a Nanodrop Lite Spectrophotometer (Thermo Fisher Scientific) and its purity was assessed by the A_260_/A_280_ ratio. 600 ng of RNA were retrotranscribed to cDNA using iScript cDNA Synthesis Kit (Bio-Rad, Hercules, CA) for further use in quantitative RT-PCR (qRT-PCR).

Transcripts encoding for *TH, CHGA, CHGB,* hypoxanthine phosphoribosyltransferase 1 (*HPRT1)* and glyceraldehyde-3-phosphate dehydrogenase *(GAPDH)* genes were measured by TaqMan qRT-PCR with PerfeCTa FastMix II Low ROX (QuantaBio) in a QuantStudio five qPCR System (Applied Biosystems). TaqMan gene expression assays employed were: Rn00562500_m1 [*TH*], Rn00572200_m1 [*CHGA*], Rn01514853_m1 [*CHGB*], Rn02042961_s1 [*SCG2*], Rn01527840_m1 [*HPRT1*] and Rn01775763_g1 [*GAPDH*] (ThermoFisher Scientific). The level of target mRNA was estimated by relative quantification using the 2^−ΔΔCt^ method and *GAPDH and HPRT1* as housekeeping genes. All reactions were performed in technical triplicates.

### Western blotting

PC12 in suspension, were incubated with 100 nM of DEX for 24, 48, 72 and 96 h. At 48 h the supplemented RPMI 1640 medium was replaced, for each time the cells were centrifugated at 600 g for 5 min. Cells were lysed for 20 min at 4 °C in TENT lysis solution containing the protease inhibitor mixture cOmplete (11697498001, Roche Diagnostics, Mannheim, Germany). Equivalent amounts of proteins (20 μg) were separated by sodium dodecyl sulfate-polyacrylamide gel electrophoresis (SDS-PAGE) using 10% acrylamide gels. The protein samples were then electroblotted onto 0.45 μm polyvinylidene difluoride membranes (Immobilon-P IPVH00010, Millipore Corporation, Billerica, MA, USA). Immunoblotting of the protein samples was performed using primary antibodies against anti-rabbit CgA (Santa Cruz Biotechnology, sc-1488, RRID: AB_2276319) and the four following anti-mouse; TH (Sigma-Aldrich, T1299, RRID: AB_477560), actin (Sigma-Aldrich, A3853, RRID: AB_262137), alpha-tubulin (Sigma-Aldrich, T6074, RRID: AB_477582), CgB (Santa Cruz Biotechnology, sc-517541, RRID: AB_2891095). The secondary antibodies used were horseradish peroxidase-conjugated anti-rabbit IgG (NA934, RRID: AB_772206), secondary horseradish peroxidase-conjugated anti-mouse IgG (NA931, RRID: AB_772210) from GE Healthcare.

Protein bands were visualized using a luminescent detection reagent, Clarity Western ECL Blotting Detection Reagent (Bio-Rad, 170-5061). The protein bands were captured and analyzed using a ChemiDoc device (Bio-Rad). Image analysis was performed using Quantity One 4.6.7 software (https://www.bio-rad.com/es-es/product/quantity-one-1-d-analysis-software) (Bio-Rad).

### Immunofluorescence

PC12 cells were seeded onto 12-mm diameter glass coverslips coated with poly-D-lysine. After 24 h in culture, cells were fixed for 10 min at RT in 4% paraformaldehyde prepared in PBS. Following fixation, cells were washed three times with 0.1% Tween-20 in PBS (PBS-T) as permeabilization solution. Free aldehyde groups were quenched with 100 mM glycine in PBS. Cells were then blocked for 1 h at RT in 5% bovine serum albumin prepared in permeabilization solution. After washing with PBS, cells were incubated overnight at RT with primary antibody (anti-goat CgA (Santa Cruz Biotechnology, sc-1488, RRID: AB_2276319, anti-mouse CgB (Santa Cruz Biotechnology, sc-517541, RRID: 2891095 and anti-mouse synaptobrevin2 (Synaptic Systems, 104-211, RRID: 887811) diluted in PBS-T. Fluorophore-conjugated secondary antibodies (Alexa fluor 488 Donkey anti-mouse, Jackson ImmunoResearch 715-545-150, RRID: AB_2340846, Alexa Fluor 568 donkey anti-goat, Thermo Fisher Scientific, A11057, RRID: AB_2534104), diluted in the same buffer, were applied for 1 h at RT. Coverslips were washed several times with PBS at RT prior to mounting. Coverslips were mounted using Fluoroshield antifade mounting medium (Sigma). High-resolution images were acquired from x–y mid-sections using an inverted microscope (Axiovert 200M, Zeiss) equipped with a 100× oil-immersion objective (α Fluar, NA 1.45, Zeiss). Immersion oil (n = 1.518, Zeiss) was applied between the objective and the coverslip. Fluorescence excitation was provided by a mercury (Hg) lamp. Excitation light was filtered using a 575/50 band-pass filter (Chroma Technology), and emitted fluorescence was collected through an HP625/50 emission filter (Chroma Technology). Images were captured with a digital EM-CCD camera (C9100-13, Hamamatsu Photonics) using HCImage software (Hamamatsu Photonics) with an exposure time of 100 ms.

### Image analysis

Image analysis was performed using MetaMorph software (Molecular Devices). Images were first processed using a flat-field background correction algorithm to reduce uneven illumination. A grayscale intensity threshold (inclusive threshold) was then applied uniformly across all images within each experiment to generate a grey level mask for spot detection. Bright punctate structures corresponding to CgA-positive vesicles were automatically identified, with detection parameters set to ensure that each spot was resolved as an isolated, single punctum. For each detected punctum, a circular region of interest (ROI) with a fixed diameter of 0.7 μm was generated centered on the punctum. These ROI coordinates were then applied to the corresponding VAMP2 and CgB channel images to identify puncta at the same spatial locations. For each ROI, the software calculated the mean fluorescence intensity within the region. Co-localization was considered positive when the mean intensity within the ROI exceeded the local background mean plus three standard deviations, in both channels. Only the mean fluorescence values of CgA-positive structures that co-localized with VAMP2 or CgB were retained for subsequent quantitative analysis.

### Amperometry

#### On-line amperometry

Cells were incubated during 48 h with and without DEX 100 nM. On the day of experiment, seven million PC12 cells were seeded onto 90 mm diameter plastic Petri dishes. Subsequently, the cells were gently detached from the dish bottom using a cell scraper, centrifuged at 600×*g* for 5 min, and resuspended in 1 ml K-H buffer for a second centrifugation under the same conditions to ensure the removal of residual drugs. Cells were loaded into a 0.22 μm filter, which served as a cell chamber (Millex SLGS0033SS, Millipore). The cell chamber was then perifused with K-H solution at 37 °C with a flow rate of 1 ml/min. The perifusate eluted from the cell chamber was directed to an electrochemical detector with a fixed potential of +650 mV (LC-4B, BioAnalytical Systems). The detector was connected to data acquisition hardware (PowerLab 8/30, ADInstruments Ltd) which provides real-time signal recording. Secretion was triggered by pulses of 70 mM KCl every 3 min. High -K^+^ (70 mM) solution was prepared by substituting NaCl for an equal amount of KCl in the K-H to maintain isotonicity of the solution. The resulting 70 mM KCl evokes secretion events were quantified by integrating the amperometric peak (AUC from each peak). The method was adapted from previously described protocols (20, 55).

### Single cell amperometry

On the day prior to the experiment, control or treated with DEX PC12 cells (5 × 10^5^) were seeded in 24-well plates containing poly-D-lysine treated 12 mm glass coverslips and cultured them in supplemented RPMI-1640 medium. Carbon fiber microelectrodes of 6 μm radius (Thornel P-55; Amoco Corp.), were prepared as described (56) and calibrated to assure the reproducibility of results (57, 58). Glass coverslips with attached PC12 cells were placed under an inverted microscope (Olympus IX51, using a 40X/0.60 NA objective). A single PC12 cell were selected and position at the center of the field of view. The carbon fiber microelectrode was gently placed onto the cell surface and the secretion was elicited with 5 s pressure ejections of 5 mM BaCl_2_ through a pipete position to 40 microns away (42). Electrochemical recordings were performed using a VA-10X potentiostat (NPI Electronics) connected to a data acquisition system (PowerLab 8/30, ADInstruments). All amperometric recordings were acquired at 4 kHz and data was filtered at 1 kHz using a low-pass filter. Cells were maintained, at 37 °C during the recordings. The detection limit, in our recordings was determinated by Faraday Laws using the threshold of amperometry noise and was calculates as approximately 25 zmol, similar to the previously reported by the Ewing group (14).

### Amperometric data analysis

Data analysis was carried out using IGOR-Pro (Wavemetrics). Tailored macros and routines were used to extract the following parameters from each spike: Imax, maximum oxidation current, expressed in pA; t_1/2_, spike width at half height, expressed in ms; Q, spike net charge, expressed in pC; m, ascending slope of spike, expressed in pA/ms (59, 60).

The data was analyzed by an investigator who was blinded to the experimental conditions. To reduce bias caused by the decaying sensitivity of an electrode over time, recordings were alternated between control and test cells; no comparisons were made between experiments carried out on different days. The discrimination threshold was fixed at 2.5 SD of the first derivative of each recording of the baseline noise. Spikes with an Imax over 2.5 pA are usually included in the data analysis. The kinetic parameters were calculated as mean values from at least 30 spikes per cell.

To avoid the deviations caused by the different number of spikes produced by each cell, the average values of spikes parameters recorded from each cell were considered as N = 1 (58, 61). All those spikes had to satisfy the following selection criteria.

i. Spikes Imax must be above the detection threshold 5 times over the SD of noise; ii. Spikes must not show overlapping; iii. None of the measured parameters can be affected by any artifacts. “n”, represent the number of independent cells in each condition were used in the statistical analysis.

### Statistics

Experiments were performed using independent biological samples, with a minimum sample size of three per group. Where applicable, samples were randomly assigned to experimental conditions, and data analysis was conducted blinded to group allocation. The exact group sizes (n) for each experimental condition are specified in the corresponding figure and table legends. No sample size calculation was performed. Data are expressed as means ± SD except Fig. 1*A* and 3*B*, where values are presented as mean ± SEM to improve data visualization. All collected data points were included in the statistical analyses, and no outliers were excluded. All data set was tested for normality using the D’Agostino-Pearson normality test. Statistical comparations between groups were performed by two-way ANOVA followed by Tukey's *post hoc* multiple-comparisons test (Figs. 1*C* and 2*B*), one-way ANOVA, with Dunnett's test (Fig. 3*D*) an unpaired Student’s *t* test with Welch´s correction (3B, 4C, 5B & D), unpaired Student’s *t* test (Fig. 3*F*), and the non-parametric Mann-Whitney rank sum test with Holm-Bonferroni correction method (Table 1). For Figure 7, *A*–*D*, the non-parametric Mann-Whitney rank sum test with Bonferroni correction was applied. For Figure 7, *E*–*H* Pairwise comparisons of regression slopes were adjusted for multiple testing using the Benjamini–Hochberg false discovery rate procedure. Differences were considered statistically significant at *p* < 0.05 (∗) and *p* < 0.01 (∗∗). Data were analyzed using Prism Software (https://www.graphpad.com) (Graphpad Software).

## Data availability

The data that support the findings of this study are available from the corresponding author upon reasonable request.

## Conflict of interest

The authors declare that they have no conflicts of interest with the contents of this article.

## References

[1] Almers W. (2003). Exocytosis. Annu. Rev. Physiol..

[2] De Camilli P., Jahn R. (1990). Pathways to regulated exocytosis in neurons. Annu. Rev. Physiol..

[3] Staal R.G.W., Mosharov E.V., Sulzer D. (2004). Dopamine neurons release transmitter via a flickering fusion pore. Nat. Neurosci..

[4] Jahn R., Lang T., Südhof T.C. (2003). Membrane fusion. Cell.

[5] Südhof T.C., Rizo J. (2011). Synaptic vesicle exocytosis. Cold Spring Harb. Perspect. Biol..

[6] Südhof T.C. (2013). Neurotransmitter release: the last millisecond in the life of a synaptic vesicle. Neuron.

[7] Edwards R.H. (2007). The neurotransmitter cycle and quantal size. Neuron.

[8] Montesinos M.S., Machado J.D., Camacho M., Diaz J., Morales Y.G., De La Rosa D.A. (2008). The crucial role of chromogranins in storage and exocytosis revealed using chromaffin cells from chromogranin A null mouse. J. Neurosci..

[9] Albillos A., Dernick G., Horstmann H., Almers W., Alvarez de Toledo G., Lindau M. (1997). The exocytotic event in chromaffin cells revealed by patch amperometry. Nature.

[10] Díaz-Vera J., Morales Y.G., Hernández-Fernaud J.R., Camacho M., Montesinos M.S., Calegari F. (2010). Chromogranin B gene ablation reduces the catecholamine cargo and decelerates exocytosis in chromaffin secretory vesicles. J. Neurosci..

[11] Pothos E., Desmond M., Sulzer D. (1996). L-3,4-dihydroxyphenylalanine increases the quantal size of exocytotic dopamine release in vitro. J. Neurochem..

[12] Kozminski K.D., Gutman D.A., Davila V., Sulzer D., Ewing A.G. (1998). Voltammetric and pharmacological characterization of dopamine release from single exocytotic events at rat pheochromocytoma (PC12) cells. Anal. Chem..

[13] Westerink R.H.S., De Groot A., Vijverberg H.P.M. (2000). Heterogeneity of catecholamine-containing vesicles in PC12 cells. Biochem. Biophys. Res. Commun..

[14] Chen T.K., Luo G., Ewing A.G. (1994). Amperometric monitoring of stimulated catecholamine release from rat pheochromocytoma (PC12) cells at the zeptomole level. Anal. Chem..

[15] White K.A., Kim B.N. (2021). Quantifying neurotransmitter secretion at single-vesicle resolution using high-density complementary metal–oxide–semiconductor electrode array. Nat. Commun..

[16] Pothos E.N., Przedborski S., Davila V., Schmitz Y., Sulzer D. (1998). D2-Like dopamine autoreceptor activation reduces quantal size in PC12 cells. J. Neurosci..

[17] Pothos E.N., Davila V., Sulzer D. (1998). Presynaptic recording of Quanta from midbrain dopamine neurons and modulation of the quantal size. J. Neurosci..

[18] Dominguez N., Estevez-Herrera J., Borges R., Machado J.D. (2014). The interaction between chromogranin A and catecholamines governs exocytosis. FASEB J..

[19] Camacho M., Machado J.D., Montesinos M.S., Criado M., Borges R. (2006). Intragranular pH rapidly modulates exocytosis in adrenal chromaffin cells. J. Neurochem..

[20] Estévez-Herrera J., Domínguez N., Pardo M.R., González-Santana A., Westhead E.W., Borges R. (2016). ATP: the crucial component of secretory vesicles. Proc. Natl. Acad. Sci. U. S. A..

[21] Moriyama Y., Hiasa M., Sakamoto S., Omote H., Nomura M. (2017). Vesicular nucleotide transporter (VNUT): appearance of an actress on the stage of purinergic signaling. Purinergic Signal.

[22] Troyer K.P., Wightman R.M. (2002). Temporal separation of vesicle release from vesicle fusion during exocytosis. J. Biol. Chem..

[23] Wightman R.M., Jankowski J.A., Kennedy R.T., Kawagoe K.T., Schroeder T.J., Leszczyszyn D.J. (1991). Temporally resolved catecholamine spikes correspond to single vesicle release from individual chromaffin cells. Proc. Natl. Acad. Sci. U. S. A..

[24] Westerink R.H.S., Ewing A.G. (2008). The PC12 cell as model for neurosecretion. Acta Physiol..

[25] Winkler H., Fischer-Colbrie R. (1992). The chromogranins A and B: the first 25 years and future perspectives. Neuroscience.

[26] Gong L.W., de Toledo G.A., Lindau M. (2007). Exocytotic catecholamine release is not associated with cation flux through channels in the vesicle membrane but Na+ influx through the fusion pore. Nat. Cell Biol..

[27] Reigada D., Díez-Pérez I., Gorostiza P., Verdaguer A., De Aranda I.G., Pineda O. (2003). Control of neurotransmitter release by an internal gel matrix in synaptic vesicles. Proc. Natl. Acad. Sci. U. S. A..

[28] Sombers L.A., Maxson M.M., Ewing A.G. (2005). Loaded dopamine is preferentially stored in the halo portion of PC12 cell dense core vesicles. J. Neurochem..

[29] Hodel A. (2001). Effects of glucocorticoids on adrenal chromaffin cells. J. Neuroendocrinol..

[30] Choi A.Y., Fukui H., Perlman R.L. (1995). Glucocorticoids enhance histamine-evoked catecholamine secretion from bovine chromaffin cells. J. Neurochem..

[31] Kim K.-T., Park D.H., Joh T.H. (1993). Parallel up-regulation of catecholamine biosynthetic enzymes by dexamethasone in PC12 cells. J. Neurochem..

[32] Elhamdani A., Brown M.E., Artalejo C.R., Palfrey H.C. (2000). Enhancement of the dense-core vesicle secretory cycle by glucocorticoid differentiation of PC12 cells: characteristics of rapid exocytosis and endocytosis. J. Neurosci..

[33] Mosharov E.V., Gong L.W., Khanna B., Sulzer D., Lindau M. (2003). Intracellular patch electrochemistry: regulation of cytosolic catecholamines in chromaffin cells. J. Neurosci..

[34] Mosharov E.V., Larsen K.E., Kanter E., Phillips K.A., Wilson K., Schmitz Y. (2009). Interplay between cytosolic dopamine, calcium, and α-Synuclein causes selective death of Substantia Nigra neurons. Neuron.

[35] Sabban E.L., Tillinger A., Nostramo R., Serova L. (2012). Stress triggered changes in expression of genes for neurosecretory granules in adrenal medulla. Cell. Mol. Neurobiol..

[36] Tischler A.S., Perlman R.L., Morse G.M., Sheard B.E. (1983). Glucocorticoids increase catecholamine synthesis and storage in PC 12 pheochromocytoma cell cultures. J. Neurochem..

[37] Fischer-Colbrie R., Wohlfarter T., Schmid K.W., Grino M., Winkler H. (1989). Dexamethasone induces an increased biosynthesis of chromogranin A in rat pituitary gland. J. Endocrinol..

[38] Rozansky D.J., Wu H., Tang K., Parmer R.J., O’Connor D.T. (1994). Glucocorticoid activation of chromogranin A gene expression. Identification and characterization of a novel glucocorticoid response element. J. Clin. Invest..

[39] Tao-Cheng J.H., Eident L.E. (1997). The vesicular monoamine transporter VMAT2 and vesicular acetylcholine transporter VAChT are sorted to separate vesicle populations in PC12 cells. Adv. Pharmacol..

[40] Pothos E.N., Larsen K.E., Krantz D.E., Liu Y., Haycock J.W., Setlik W. (2000). Synaptic vesicle transporter expression regulates vesicle phenotype and quantal size. J. Neurosci..

[41] Kim T., Tao-Cheng J.H., Eiden L.E., Loh Y.P. (2001). Chromogranin A, an “on/off” switch controlling dense-core secretory granule biogenesis. Cell..

[42] Baraibar A.M., de Pascual R., Camacho M., Domínguez N., David Machado J., Gandía L. (2018). Distinct patterns of exocytosis elicited by Ca 2+ , Sr 2+ and Ba 2+ in bovine chromaffin cells. Pflugers Arch..

[43] Machado J.D., Gómez J.F., Betancor G., Camacho M., Brioso M.A., Borges R. (2002). Hydralazine reduces the quantal size of secretory events by displacement of catecholamines from adrenomedullary chromaffin secretory vesicles. Circ. Res..

[44] Guérineau N.C. (2024). Adaptive remodeling of the stimulus-secretion coupling: lessons from the ‘stressed’ adrenal medulla. Vitam. Horm..

[45] Carbone E., Borges R., Eiden L.E., García A.G., Hernández-Cruz A. (2019). Chromaffin cells of the adrenal medulla: physiology, pharmacology, and disease. Compr. Physiol..

[46] Qin N., Chen Z., Xue R. (2022). A two-subpopulation model that reflects heterogeneity of large dense core vesicles in exocytosis. Cell Cycle.

[47] Mark Wightman R., Domínguez N., Borges R. (2018). How intravesicular composition affects exocytosis. Pflugers Arch..

[48] Borges R., Díaz-Vera J., Domínguez N., Arnau M.R., MacHado J.D. (2010). Chromogranins as regulators of exocytosis. J. Neurochem..

[49] Videen J.S., Mezger M.S., Chang Y.M., O’Connor D.T. (1992). Calcium and catecholamine interactions with adrenal chromogranins. Comparison of driving forces in binding and aggregation. J. Biol. Chem..

[50] Díaz-Vera J., Camacho M., Machado J.D., Domínguez N., Montesinos M.S., Hernández-Fernaud J.R. (2012). Chromogranins A and B are key proteins in amine accumulation, but the catecholamine secretory pathway is conserved without them. FASEB J..

[51] Helle K.B., Reed R.K., Pihl K.E., Serck-Hanssen G. (1985). Osmotic properties of the chromogranins and relation to osmotic pressure in catecholamine storage granules. Acta Physiol. Scand..

[52] Pothos E.N., Mosharov E., Liu K.P., Setlik W., Haburcak M., Baldini G. (2002). Stimulation-dependent regulation of the pH, volume and quantal size of bovine and rodent secretory vesicles. J. Physiol..

[53] Erickson J.D., Schäfer M.K.H., Bonner T.I., Eiden L.E., Weihe E. (1996). Distinct pharmacological properties and distribution in neurons and endocrine cells of two isoforms of the human vesicular monoamine transporter. Proc. Natl. Acad. Sci. U. S. A..

[54] Pereira D.B., Sulzer D. (2012). Mechanisms of dopamine quantal size regulation. Front. Biosci. (Landmark Ed).

[55] González-Santana A., Estévez-Herrera J., Seward E.P., Borges R., Machado J.D. (2021). Glucagon-like peptide-1 receptor controls exocytosis in chromaffin cells by increasing full-fusion events. Cell Rep..

[56] Kawagoe K.T., Zimmerman J.B., Wightman R.M. (1993). Principles of voltammetry and microelectrode surface states. J. Neurosci. Methods.

[57] Machado J.D., Montesinos M.S., Borges R. (2008). Good practices in single-cell amperometry. Methods Mol. Biol..

[58] Machado J.D., Montenegro P., Domínguez N. (2023). Quantal release analysis of electrochemically active molecules using single-cell amperometry. Methods Mol. Biol..

[59] Machado J.D., Segura F., Brioso M.A., Borges R. (2000). Nitric oxide modulates a late step of exocytosis. J. Biol. Chem..

[60] Segura F., Brioso M.A., Gómez J.F., Machado J.D., Borges R. (2000). Automatic analysis for amperometrical recordings of exocytosis. J. Neurosci. Methods.

[61] Colliver T.L., Hess E.J., Pothos E.N., Sulzer D., Ewing A.G. (2000). Quantitative and statistical analysis of the shape of amperometric spikes recorded from two populations of cells. J. Neurochem..

